# Magnetic torsional actuation of carbon nanotube yarn artificial muscle†

**DOI:** 10.1039/c8ra01040d

**Published:** 2018-05-11

**Authors:** Duck Weon Lee, Shi Hyeong Kim, Mikhail E. Kozlov, Xavier Lepró, Ray H. Baughman, Seon Jeong Kim

**Affiliations:** Center for Self-Powered Actuation, Department of Biomedical Engineering, Hanyang University Seoul 04763 Korea sjk@hanyang.ac.kr; The Alan G. MacDiarmid NanoTech Institute, University of Texas at Dallas Richardson TX 75083 USA

## Abstract

Magnetically driven torsional actuation of a multiwalled carbon nanotube (MWNT) yarn was realized by first biscrolling NdFeB magnetic particles into helical yarn corridors to make a magnetic MWNT yarn. The actuating device comprised a pristineMWNT yarn that was connected to the magnetic MWNT yarn, with a paddle attached between these yarns. The application of a magnetic field reversibly drove torsional actuation of up to 80° within ∼0.67 seconds. This magnetic actuator was remotely powered, and its actuation stroke was the same when the muscle array was at 20 °C and at −100 °C.

## Abstract 2

Magnetically driven torsional actuation of a multiwalled carbon nanotube (MWNT) yarn was realized by first biscrolling NdFeB magnetic particles into helical yarn corridors to make a magnetic MWNT yarn.

## Graphical abstract

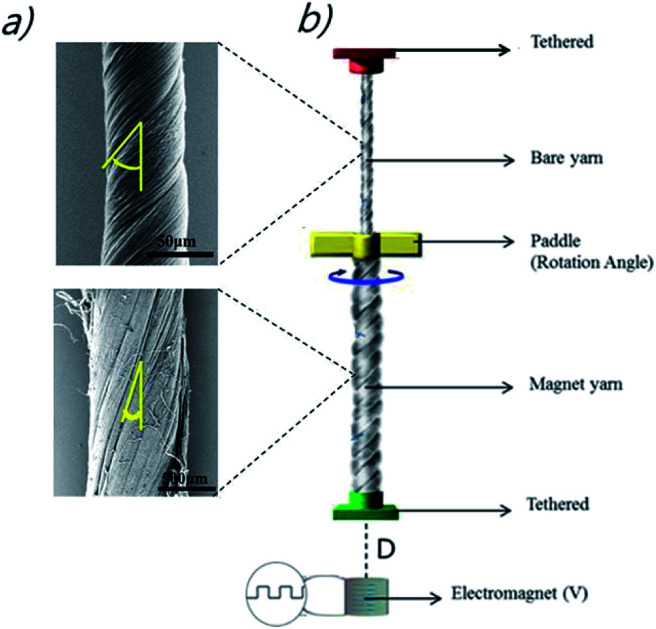

## A. Introduction

Various types of torsional artificial muscles based on twisted carbon multiwalled nanotube (MWNT) yarns have recently been reported in the literature.^1–7^ For example, electrochemically charging a pristine-twist-spun yarn immersed in a liquid electrolyte^1^ and electrothermally heating a twist-spun wax-infiltrated hybrid MWNT yarn^2^ results in high angle torsional muscle rotation. In particular, a wax-infiltrated MWNT yarn provided a torsional rotation speed of 11 500 rpm, a maximum torsional stroke of 71.2° per millimeter of yarn length, a maximum torque-to-mass ratio of 8.42 N m kg^−1^, and millions of reversible cycles.^2^ These torsional actuations originate from the volumetric expansion of the twisted yarn structure, which are caused by the electrochemical insertion of electrolyte ions for electrochemical actuation and thermal expansion of a guest material for thermal actuation.^1,2^

This paper here presents a means for torsionally actuating carbon nanotube (CNT) yarn muscles that does not require electrochemical processes (and the associated electrolyte and counter electrode)^1,3^ liquid or vapour absorption^2,5,6^ or yarn heating.^2,4^ The presented actuation results from applying an external magnetic field along the yarn direction of a CNT yarn that contains magnetic particles. Consequently, the yarn actuator is driven remotely by an electromagnetic field, so no yarn heating or electrolyte is needed.^8,9^ As such, this actuation method can be applied over extreme temperatures in vacuum, air, explosive, flammable, or corrosive environments, as long as the environment does not degrade either the properties of the CNT yarn or those of the magnetic particles in the CNT yarn.

## B. Experimental section

**Fig. 1:**
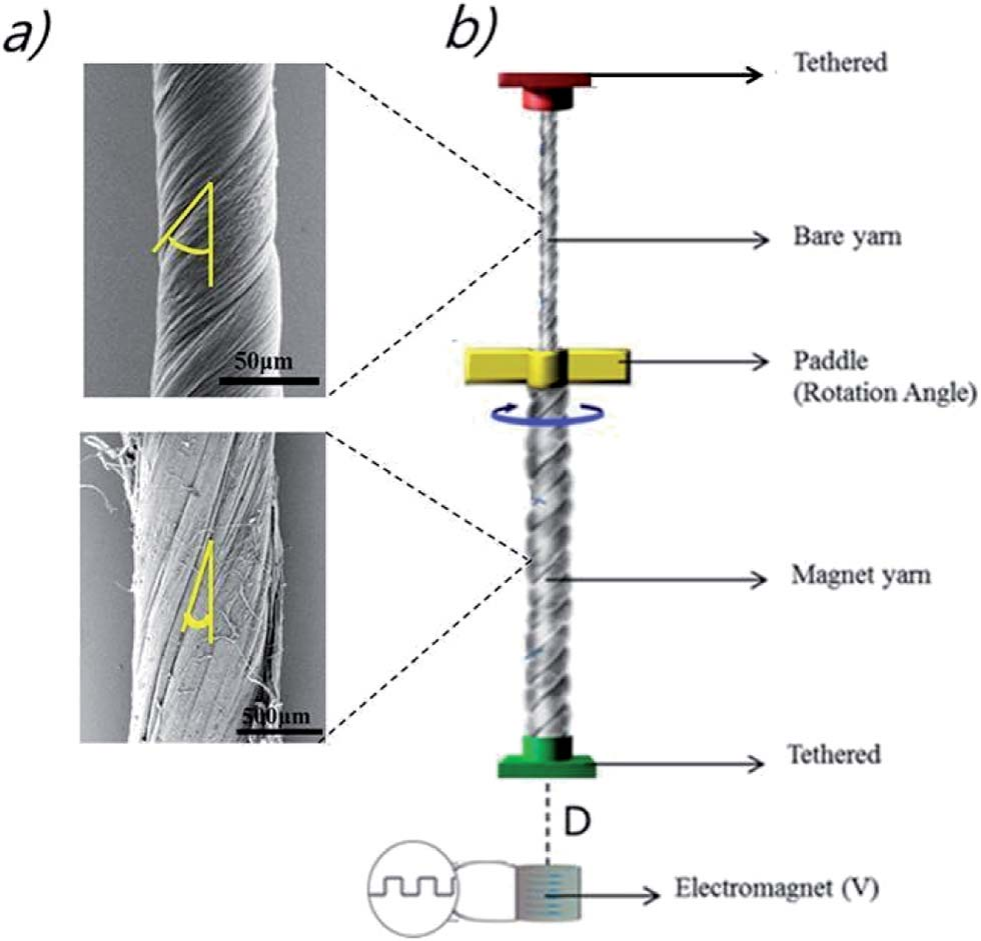
(a) SEM images of pristine and magnetic particle filled MWNT yarns, where the yellow lines on the images define the bias angle for each yarn, (b) schematic diagram showing the configuration of the magnetically actuated, twist-spun MWNT yarn.

In the explored design, both pristine and magnetic yarns were twisted to provide the same handedness of inserted twist and joined together with a paddle at their interconnection. Also, this yarn array was suspended from an upper tether that prohibited end rotation and translation and connected on the bottom end of the magnetic yarn to a torsionally tethered 10.7 mg plastic weight, as shown in Fig. 1b. This paddle enabled characterization of torsional muscle actuation. The bottom end of the magnetic yarn was attached to the non-magnetic weight, which was restricted from rotating, but free to move in the vertical direction as the artificial muscles contracted and expanded. The muscle was actuated by the magnetic field of an electromagnet, which was positioned 1 to 8 mm below the end of the magnetic yarn segment (D) in Fig. 1b (Movie S1†). The rotation of the paddle and the vertical displacement of the bottom end of the muscle were recorded by a digital camera (Sony Nex-7). The measurements were performed at room temperature and at temperatures down to −100 °C. In the latter case, the muscle was placed in an environmental chamber and cooled with liquid nitrogen vapor, while the yarn temperature was recorded by a thermocouple placed close to the yarn.

The electromagnet driving the actuation was 1.4 cm high, and had internal and external diameters of 0.6 cm and 1.5 cm, respectively. This electromagnet was wound from ∼6300 turns of 10 micron-diameter insulated copper wire and the maximum electric current was about 0.4 A at 40 V. The torsional actuator stroke was varied by changing the magnetic field that drove actuation and this field was varied by changing the voltage provided by an Agilent E3613A DC power supply (Movie S2 and S3†). Furthermore, liquid nitrogen (N) was used to lower actuator temperature to below −100 °C, in order to demonstrate that operation temperature range of this actuator was broader than that of any other torsional actuators.

The pristineMWNT yarn (with *Z* direction twist) was obtained by inserting twist into a stack of ribbon-shaped MWNT sheets that were drawn from a MWNT forest, which was grown on a silicon substrate by using the chemical vapour deposition (CVD) of acetylene gas.^10,11^ Transmission and scanning electron microscope images indicate that the MWNTs in the ∼350 μm high forests have an outer diameter of ∼9 nm, contain ∼6 walls and form large bundles. The amount of non-combustible material in the drawn nanotubes is below 1 wt%, which places an upper limit on the amount of the residual iron catalyst used for CVD. Upon inserting 7960 turns per m of twist into a stack of five sheet ribbons (where the inserted twist is with respect to the ribbon length), a pristine-twisted CNT yarn, having a diameter of about 40 μm and a length of 2.7 cm, was obtained. The bias angle of the pristine-yarn, which was the angle between the nanotubes on the yarn surface and the yarn direction, was 45°.

**Fig. 2:**
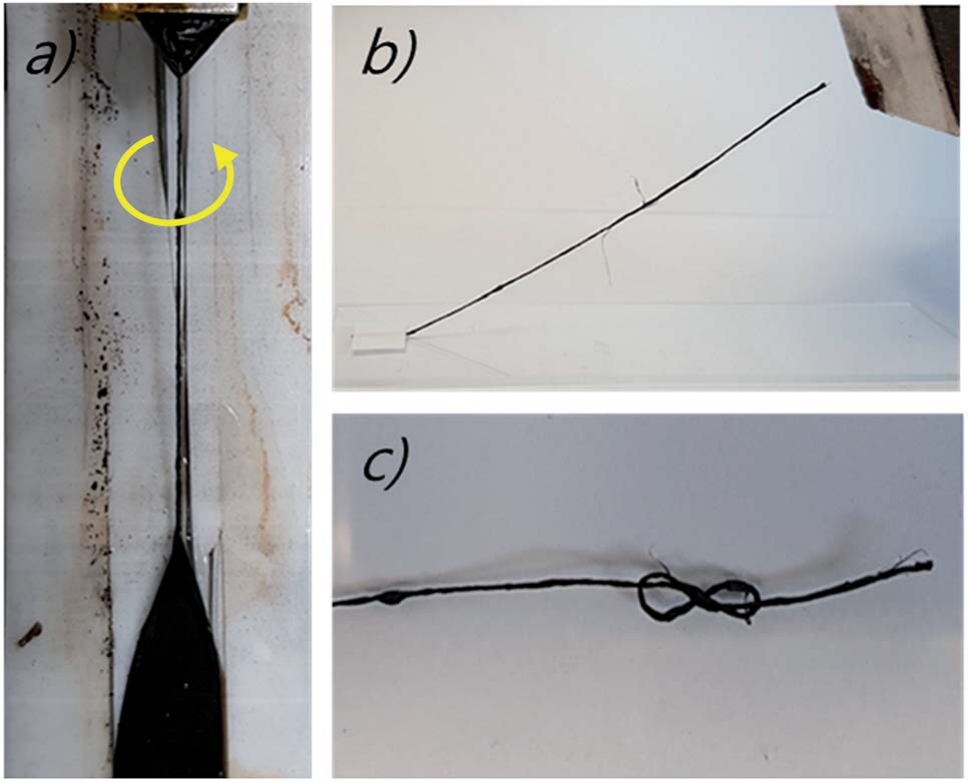
(a) Optical images showing the magnetic ribbon-shaped MWNT sheet is being fabricated by the biscrolling method,^1^ (b) the attraction of the magnet yarn to a magnet, and (c) a knotted magnetic-particle-filled MWNT yarn.

The method used to fabricate the magneticMWNT yarn (*Z* direction) is as follows: the magnetic MWNT yarn contains NdFeB particles, which were obtained by grounding commercially available block-type bulk magnets (http://www.magnetkingdom.com, MK-0023) into hundred-nanometer-to-micrometer size particles by using a mortar. These particles were incorporated in the corridors of a twist-spun CNT yarn using the biscrolling process as shown in Fig. 2a.^1,8,12^ In using this biscrolling process, a stack of up to 75 alternating layers of the MWNT sheet strips was assembled on Teflon tape, wherein the NdFeB particles were manually deposited on a sheet layer before the next sheet layer was attached to the substrate. A surface-tension-based densification process was deployed to increase stack density and the interaction between the MWNT sheets and the magnetic particles. This densification process involves the absorption of drops of methanol on the sheet stack and subsequent evaporation of the methanol. After peeling the sheet stack from the Teflon substrate, the ends of the sheet stack were attached to rigid end supports that were used to insert 467 turns per m of twist (normalized with respect to stack length). The resulting biscrolled magnetic yarn had a diameter of 780 μm, a bias angle of 20°, and the yarn contained about 99 wt% of magnetic particles. The alignment of the magnetic particles placed in the MWNT yarn created the magnetic moment.^1,8,13,14^ The interaction between the magnetic-particle-filled MWNT yarn and a magnet, and its flexibility as shown in Fig. 2b and c, respectively. Finally, the bottom of the pristine-MWNT yarn and the top of the magnetic-MWNT yarn were interconnected by epoxy resin (DEVCON®home) and then the paddle was attached in order to indicate the rotation angle, as shown in Fig. 1b.

## C. Results

**Fig. 3:**
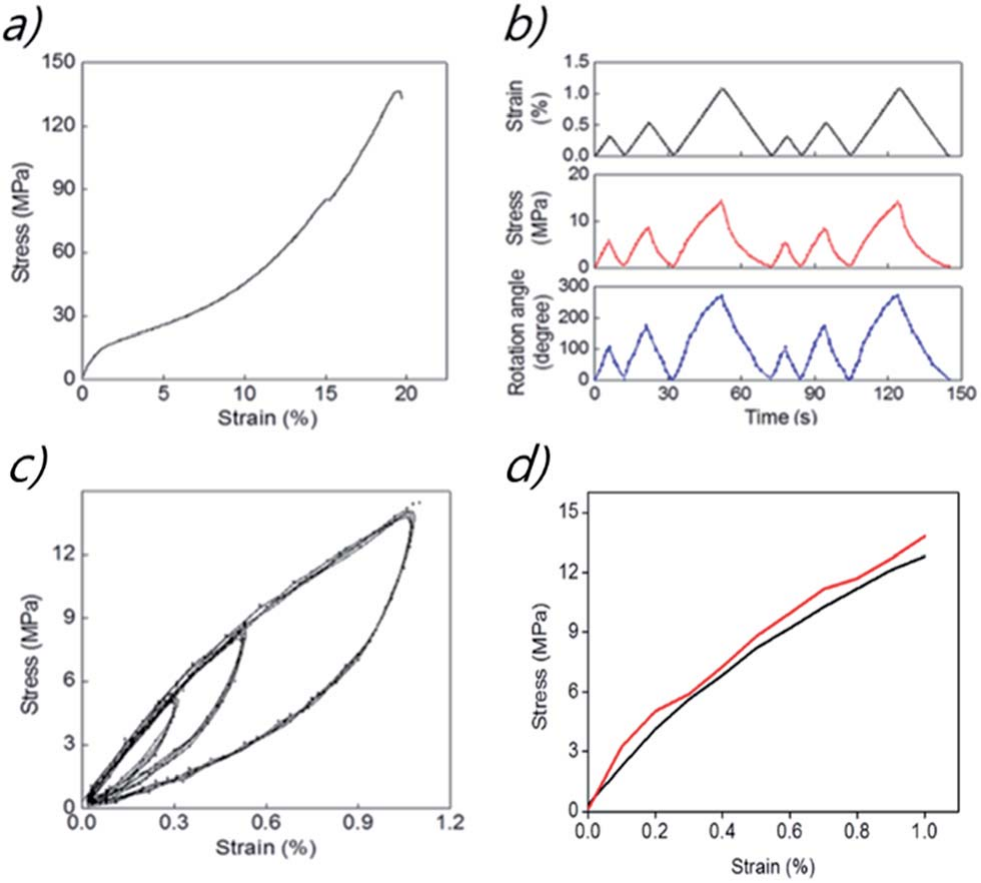
Mechanical properties of the pristineyarn and magnetic-particle-filled MWNT yarn: (a) the stress–strain curve of the yarn array up to yarn fracture, (b) the time dependence of an applied strain (top panel) and the time dependence of resulting stress (middle panel) and resulting torsional rotation (bottom panel) when the 8.5 cm-long linear muscle array was stretched at 10 μm s^−1^. (c) Strain–stress curve showing hysteresis during loading and unloading for the low-strain elastic region. (d) The dependence of stress on applied strain for the low-strain elastic region in (a) (red line) and from the time dependent results of (b) (black line) for another identically prepared sample, using maximum stresses and strains.

The yarn actuator was made of pristine yarn on the top and a magnet yarn containing NdFeB on the bottom. The mechanical properties of torsionally tethered linear array of the yarn actuator were measured to provide results shown in Fig. 3a where stresses are normalized with respect to the magnetic-particle-filled yarn and strains are with respect to the total initial length of the array. Fig. 3a shows the stress–strain curve up to yarn fracture for the torsionally tethered linear array of pristine-yarn and magnetic-particle-filled MWNT yarn segments with the attached paddle. The lengths of the pristine-yarn and the magnetic yarn in this array are 2.7 cm and 5.8 cm, respectively, and the moment of inertia of the 12.5 mg weight paddle (which is 1.5 cm long and 0.5 cm wide) is 2.34 mg cm^2^. Considering that the magnetic yarn contains about 99 wt% magnetic particles, the observed tensile strength of the yarn array (after removing the paddle) is remarkably high (about 136 MPa), and the fracture strain is up to 19%. This segmented yarn array deforms irreversibly unless the applied strain is below about 1%.

Fig. 3b shows the dependence of stress and paddle rotation on applied strain for the strain region that provides reversible behaviour. As indicated here, the application of a tensile strain of 1.05% (normalized to the entire 8.5 cm length of the interconnected pristine and magnetic yarns) resulted in a paddle rotation of 280° (corresponding to 3.29 turns per mm, when normalized to the total length of pristine and magneticyarns). Stretching the pristineyarn and magnetic-particle-filled MWNT yarn array caused the magnetic yarn to decrease twist and the pristineyarn to correspondingly increase twist. As will be discussed later, these results suggest that the observed magnetically-induced paddle rotation is being produced by the tensile stress on the yarn that results from the attraction of the magnetic yarn segment to the electromagnet.

Fig. 3c shows the dependence of stress on applied strain for the pristine-yarn and magnetic-particle-filled MWNT yarn linear array is highly hysteretic in the low strain reversible region, as it is already documented for the pristineyarn. At significantly higher tensile strains, deformation becomes irreversible. The strain–stress curves indicate that the pristineyarn and magnetic-particle-filled MWNT yarn behave visco-elastically, and provide large area hysteresis loops. The elastic yarn energy in these areas is dissipated because of internal friction between individual carbon nanotubes. The area of hysteresis loops increase with increasing magnitude of applied strain.^15,16^ Moreover, each hysteresis loop is highly reversible, which means that the initial point is always the same as the final point, regardless of the magnitude of the strain when a different tensile strength is applied. Hence, the hysteresis loops of the magnetic yarns and their reversibility are very stable for strains in the elastic deformation regime. This reversible mechanical performance is important for our realizing reversible magnetically-driven actuation.

Fig. 3d shows the agreement between the dependence of stress on strain derived from the stress–strain curve of Fig. 3a and the maximum stress and maximum strains for the time dependent results of Fig. 3d, which are for a different (but identically prepared) linear array of the pristine-yarn and magnetic-particle-filled MWNT yarn.

**Fig. 4:**
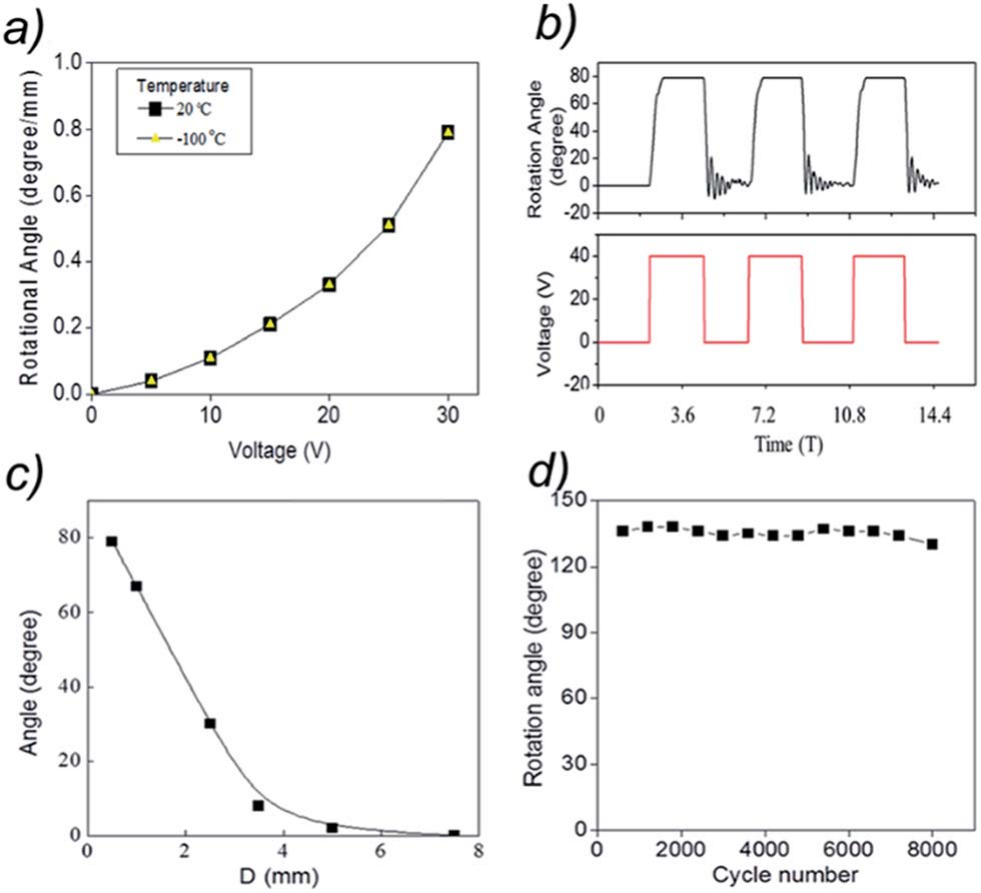
Actuation results for the linear array of pristine and magnetic-MWNT yarns: (a) the dependence of length-normalized torsional stroke at 20 °C (black) and at −100 °C (yellow) on the voltage applied to the electromagnet. (b) The torsional actuation resulting from the application of a 40 V, 0.25 Hz square wave voltage to the electromagnet. (c) The dependence of maximum torsional rotation angle on the separation between the bottom end of the magnetic yarn and the electromagnet (<40 V), (d) demonstration that there is little effect of on maximum torsional rotation on cycling by applying a 0.25 Hz, square-wave tensile strain of 0.3% for 8000 cycles.

Fig. 4a shows that the torsional rotation of the paddle quadratically depends upon the voltage applied to the electromagnet. As for the case of mechanically stretching the yarn array, applying the external magnetic field to the pristineyarn and magnetic-particle-filled MWNT yarn caused the magnetic yarn to decrease twist and the pristineyarn to correspondingly increase twist.^13–15^ Importantly, note that torsional actuation is independent of temperature from room temperature to −100 °C (yellow triangle points) to room temperature, which provides an advantage over thermally powered artificial muscles based on hybrid CNT (carbon nanotube) yarn, as well as electrochemical CNT muscles.

Fig. 4b pictures the time dependence of the torsional rotation that was obtained by applying a 0.25 Hz square-wave voltage to the electromagnet. This data, which was obtained by frame-by-frame analysis of a video recording of paddle rotation, shows the reversibility of torsional actuation. The maximum rotation of 79° (corresponding to a magnetically-induced torsional stroke of 0.79° mm^−1^) occurs within ∼0.67 seconds when 40 V is applied to the electromagnet. In addition, it is reversed in ∼0.24 seconds when the voltage switches to 0 V, providing thereafter small amplitude torsional oscillations that decay in ∼2.3 seconds. Oscillatory decay of torsional stroke to the unactuated rotation angle, as well as oscillatory approach to the maximum actuated angle, can also be found testing thermally powered wax-infiltrated yarns.^2,4^ However, during actuation of an electromagnetically actuated yarn an oscillation before reaching the maximum torsional stroke is not detected.

Fig. 4c shows the dependence of torsional stroke on the distance (*D*) between the lower end of the magnetic yarn and the top of the electromagnet, when 40 V was applied to the electromagnet. Note than the torsional stroke linearly increases with decreasing *D* (from 8° for *D* = 3.5 mm to 79° for *D* = 0.4 mm). Larger values of *D* provide a smaller dependence of torsional stroke on *D*, until no significant torsional actuation is seen for *D* = 7.5 mm. The durability was investigated by using another pristine-yarn and magnetic-particle-filled MWNT yarn to test the change in the rotation angle of the paddle for the MWNT yarn after 8000 cycles. The maximum rotation angle is consistently maintained at approximately 135° at 0.25 Hz (Keithley sourcemeter) as shown in Fig. 4d. The results demonstrate that stretch-driven torsional actuation of the pristine- and magnetic-MWNT yarn array at 0.3% strain does not provide significant degradation in torsional stroke over 8000 stretch–release cycles.

How does this magnetically driven torsional muscle operate? Application of the magnetic field along the muscle-length provides a tensile stress on the muscle, which increases with increasing *D* from the bottom end of the magnetic yarn and then reaches a plateau which is reached close to the bottom of the magnetic yarn, which is indicated from the results in Fig. 4c. The magnetic field causes a force on the magnetic particles which transfer the force *via* friction to the MWNTs. When stretched, both the pristineyarn and magnetic-particle-filled MWNT yarn segments tend to untwist in order to help enable this elongation.^16–19^ If the magnetic yarn and the pristine-yarn segments had the same mechanical properties and same dimensions (and variation in magnetically induced stress over the bottom pre-plateau region of the magnetic yarn were ignored), no paddle rotation would occur (since the torques applied to the paddle by the neat yarn and magnetic yarn would be oppositely directed, and thereby cancel). However, largely due to the much larger diameter (786 μm) of the magnetic yarn, compared with that for the pristineyarn (45 μm), the torsional force constant for untwisting the magnetic yarn segment is much larger than for increasing twist in the pristineyarn.^1,8,20–22^

For 30 V applied to the electromagnet, the yarn torsional rotation angle (normalized by the entire yarn length) was 0.79° mm^−1^ (Fig. 4a). This value is much higher than the rotation angle previously reported for piezoelectric ceramics, conducting polymers, and shape-memory alloys, which are 0.008° mm^−1^,^23^ 0.01° mm^−1^,^24^ and 0.15° mm^−1^,^25^ respectively. However, this rotation angle for the magnetically driven actuator is much smaller than observed for electrochemically actuated pristine-yarns (250° mm^−1^) and for thermally powered hybrid yarn actuators (71.2° mm^−1^).^1,2^ Experimental and theoretical work indicates that torsional stroke (per actuator total length) was inversely proportional to yarn diameter when the bias angle in the yarn segments, the ratio of the length of each segment to the segment diameter, and the applied tensile stress during yarn fabrication and actuation are unchanged. These results suggested that it should be possible to increase the torsional stroke from the present 0.79° mm^−1^ to 7.9° mm^−1^ by scaling downward the magnetic segment diameter from 786 μm to 78.6 μm and the pristine segment diameter from 40 μm to 4.0 μm.

Only the magnetically powered torsional muscles operate as well at −100 °C as at room temperature (and can likely operate at far lower temperatures) by a remote control. In addition, these muscles will work at high temperatures in gaseous environments that do not cause reactions of the magnetic particles or the carbon nanotubes, as long as the magnetic properties of the magnetic particles are unaffected.

**Fig. 5:**
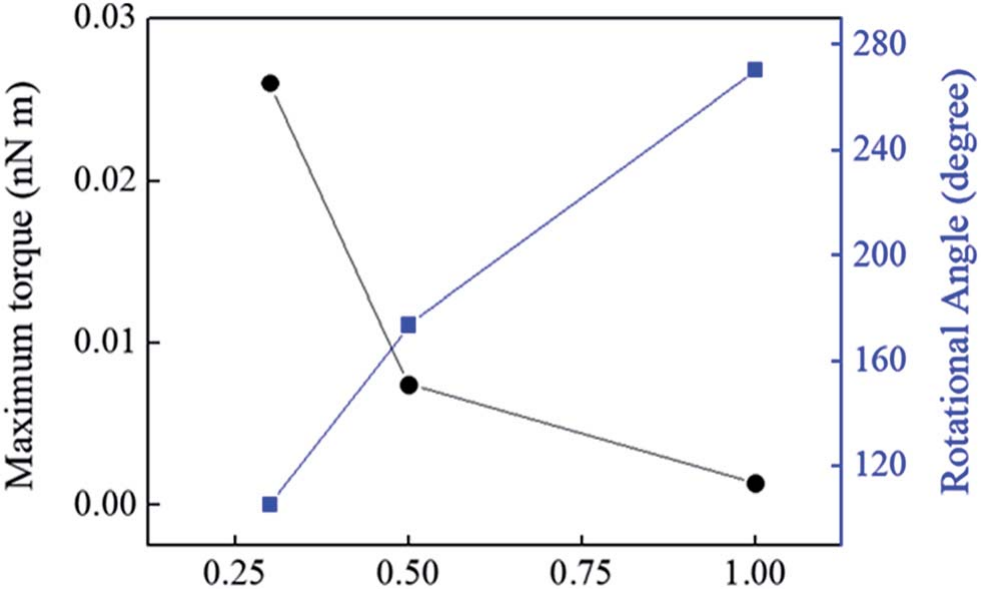
Change in maximum torque (nN m) and the rotational angle (degree) *versus* strain (%).

In addition, by using the Instron, value of maximum torque was obtained based on change in strain (%) at constant speed with rotational angle (degree) as shown in the Fig. 5. Although the maximum torque was very low because it was made by increasing the constant speed of the Instron. However, the maximum torque could be up to 5.68 (nN m) with the electromagnet system at 40 V. The maximum torque was calculated by the following equationThe maximum torque (*τ*) = *Ia*where, *I* is the moment of inertia of the paddle, and *a* is an initial paddle acceleration.

## D. Conclusions

A magnetically driven torsional actuator was demonstrated that it is remotely powered and can be operated at any temperature and in any environments that does not degrade the properties of the magnetic particles or the MWNT yarns. Identical torsional stokes of 79° (corresponding to a stroke of 0.79° mm^−1^, when normalized to total yarn length) were realized at 20 °C and at −100 °C. Since the actuation mechanism is yarn stretch as a result of the application of a magnetic field, increasing the applied magnetic field would enable the torsional stroke for this particular actuator to be increased to 280° (corresponding to 3.29 turns per mm, when normalized to the total length of pristine and magneticyarns), which is the stroke that was demonstrated by muscle stretch. Scaling arguments suggest that it should be possible to increase the torsional stroke from the present 0.79° mm^−1^ to 7.9° mm^−1^ by scaling downward the magnetic segment diameter from 786 μm to 78.6 μm and the pristine segment diameter from 40 μm to 4.0 μm. The reversibility of torsional stroke during cycling was demonstrated by applying a 0.25 Hz, square-wave tensile strain of 0.3% for 8000 cycles.

## Conflicts of interest

There are no conflicts to declare.

## Supplementary Material

RA-008-C8RA01040D-s001

RA-008-C8RA01040D-s002

RA-008-C8RA01040D-s003
